# Nontoxic KBBF Family Member Zn_2_BO_3_(OH): Balance between Beneficial Layered Structure and Layer Tendency

**DOI:** 10.1002/advs.201901679

**Published:** 2019-09-16

**Authors:** Xuefei Wang, Fangfang Zhang, Le Gao, Zhihua Yang, Shilie Pan

**Affiliations:** ^1^ CAS Key Laboratory of Functional Materials and Devices for Special Environments Xinjiang Key Laboratory of Electronic Information Materials and Devices Xinjiang Technical Institute of Physics and Chemistry CAS 40‐1 South Beijing Road Urumqi 830011 China; ^2^ Center of Materials Science and Optoelectronics Engineering University of Chinese Academy of Sciences Beijing 100049 China

**Keywords:** borates, crystal growth habits, KBBF family, nonlinear optical materials, second harmonic generation

## Abstract

The conflict between beneficial layered structure for performances and layered growth habits in KBe_2_BO_3_F_2_ (KBBF) always restricts its practical applications. A beryllium‐free KBBF family member, Zn_2_BO_3_(OH), is explored to feature the same topological layer with KBBF by replacing [BeO_3_F]^5−^ with [ZnO_3_(OH)]^5−^ and excellent UV performances. It exhibits a second harmonic generation response of about 1.5 × KH_2_PO_4_ with the UV cutoff edge of 204 nm. The birefringence of Zn_2_BO_3_(OH) in the visible region is about 0.067, which is larger than those of commercial UV crystals LiB_3_O_5_, CsB_3_O_5_, and CsLiB_6_O_10_. Additionally, it has excellent thermal and water‐resistant stabilities. Owing to the removal of interlayer cations, Zn_2_BO_3_(OH) shows better growth habits than KBBF while achieving the balance between beneficial layered structure and layer tendency.

## Graphical abstract

Noncentrosymmetric Zn_2_BO_3_(OH) achieves nontoxic evolution from KBe_2_BO_3_F_2_ (KBBF) and BBF with a balance between the KBBF‐type layer and layered growth habit.

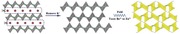

Nonlinear optical (NLO) materials have been at the forefront of laser technology due to their wide and significant applications in expanding the output wavelengths of all‐solid‐state lasers.^1^ Many crucial factors need to be considered during assessing an NLO material, including second harmonic generation (SHG) ability, optical transparency, birefringence, thermal and environmental stability, growth habit, and so forth.^2^ In the last few decades, numerous NLO materials possessing excellent ultraviolet (UV) and deep‐ultraviolet (DUV) properties were discovered.^3^ The most representative examples are β‐BaB_2_O_4_ (β‐BBO),[qv: 3a] CsLiB_6_O_10_ (CLBO),[qv: 3b] LiB_3_O_5_ (LBO),[qv: 3c] CsB_3_O_5_ (CBO)[qv: 3d] for UV and KBe_2_BO_3_F_2_ (KBBF)[qv: 3e] for DUV applications. However, their intrinsic imperfections on one or more aspects such as overlarge birefringence, hygroscopicity, layered growth tendency, or toxicity brought some troubles in practical applications. For improving the performances, several rational design strategies for discovering new UV/DUV NLO materials were proposed in recent years. One strategy is to employ extended fundamental build units like [CO_3_]^2−^,^4^ [NO_3_]^−^,^5^ [SO_4_]^2−^,^6^ and [BO*_x_*F_4−_ *_x_*]^(^ *^x^* ^+1)−^ (*x* = 1–3)^7^ besides borate groups to design new structures. Another effective approach is to modify known structures on the basis of classical UV/DUV NLO materials.

KBBF is the unique crystal that allows efficient generation of DUV light by direct frequency doubling. The special 2D layered structure of KBBF integrates many advantages such as large bandgap, large SHG responses, moderate birefringence, and so on, which is regarded as a typical prototype for designing new NLO materials.^8^ However, the layered structure of KBBF also leads to a severe layer growth habit which makes it difficult to grow large crystals. Up to now, several kinds of approaches that can effectively improve the crystal growth habit were reported: i) introducing new structural units to reinforce the interlayer bonds like Rb_3_Al_3_B_3_O_10_F^9^ and Pb_2_BO_3_X (X = Cl, Br, and I),^10^ ii) connecting layers by stronger covalent units like Cs_3_Zn_6_B_9_O_21_ 11 and CsZn_2_B_3_O_7_,^12^ iii) removing interlayer cations and connecting each layer directly like Be_2_BO_3_F.^13^ These modifications enhance the interlayered interaction and improve the crystal growth habit effectively. In recent years, beryllium‐free KBBF family materials were continuously reported considering the toxicity of BeO used in the preparation of KBBF. It has been proved that the zinc atom is an excellent substitution candidate for beryllium atom. The replacement from Be to Zn was embodied by the series of AZn_2_BO_3_X_2_ (A = Na, K, Rb, NH_4_; X = Cl, Br).^8^, ^14^ These compounds feature [Zn_2_BO_3_X_2_]_∞_ (X = Cl, Br) layers that are derived from the [Be_2_BO_3_F_2_]_∞_ layers in KBBF with the substitution of [BeO_3_F]^5−^ to [ZnO_3_X]^5−^, which provides large SHG responses and keeps moderate birefringence.

Here in this work, a zinc borate compound, namely, Zn_2_BO_3_(OH) with KBBF‐type structure was explored to successfully achieve the balance between beneficial layered structure and layer tendency. Here, we highlight that Zn_2_BO_3_(OH) exhibits a SHG response of about 1.5 times that of KH_2_PO_4_ (KDP), a UV cutoff edge of 204 nm, and a moderate birefringence for phase matching in the UV region. In addition, Zn_2_BO_3_(OH) has excellent thermal and water‐resistant stabilities that are favorable for practical applications. Specifically, Zn_2_BO_3_(OH) shows good growth habits due to the removal of interlayer cations. Based on the first principle calculations, we provide a method for assessing the growth habits from the perspective of thermodynamics, which describes the anisotropy of crystal growth more comprehensively.

Zn_2_BO_3_(OH) crystallizes in the chiral space group *P*2_1_ (No. 4) of the monoclinic system with lattice parameters *a* = 5.731(7) Å, *b* = 4.952(4) Å, *c* = 6.881(6) Å, β = 99.09(5)°, *Z* = 2. It was previously reported for other purposes but none of the works reported it as a NLO material.^15^ All B atoms are coordinated to three O atoms to form the [BO_3_]^3−^ triangles and the Zn atoms are bonded with four O atoms to form [ZnO_3_(OH)]^5−^ tetrahedra. Zn_2_BO_3_(OH) achieves nontoxic evolution and features 2D [Zn_2_BO_3_(OH)_2_]_∞_ layers that are transformed from the [Be_2_BO_3_F_2_]_∞_ layers in KBBF with the replacement from Be^2+^ to Zn^2+^ and F^−^ to [OH]^−^. **Figure** **1**a–c presents the structural transformation procedure from KBBF to *C*2‐BBF and then to Zn_2_BO_3_(OH). As is shown in these figures, *C*2‐BBF is transformed from KBBF with the removal of the K atoms. And, [Be_2_BO_3_F_2_]_∞_ layers are directly connected with adjacent layers via F—Be—F connections, which is beneficial for improving its growth habits. The improvement is also reported in γ‐BBF due to the stronger Be—F bonds than K—F ionic bonds. Since the size of [ZnO_3_(OH)]^5−^ group is larger than that of [BeO_3_F]^5−^, the [ZnO_3_(OH)]^5−^ groups “incline” with an intersection angle of 58.5° to form the pleated layers. Figure 1d,e clearly shows that both the layers have same topological frameworks, which confirms that the [Zn_2_BO_3_(OH)_2_]_∞_ layers in Zn_2_BO_3_(OH) are totally transformed from the [Be_2_BO_3_F_2_]_∞_ layers in KBBF and *C*2‐BBF. Accordingly, the [BO_3_]^3−^ groups in Zn_2_BO_3_(OH) show interleaved arrangements while keeping nearly parallel with the same orientation in KBBF (see Figure 1f), which is beneficial for large SHG intensity and birefringence. Through sharing the mutual O4 atom in [OH]^−^ of two [ZnO_3_(OH)]^5−^ tetrahedra in adjacent layers, each layer is connected via relatively strong Zn—O bonds with shorter interlayer space, which is expected to overcome the layer habit during the practical crystal growth. According to the subsequent observation with the microscope, the title compound shows better growth habits.

**Figure 1:**
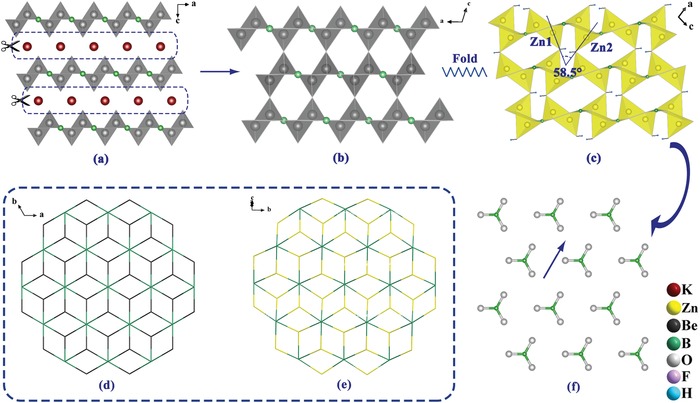
Structural transformation from KBBF to *C*2‐BBF to Zn_2_BO_3_(OH). The structure of a) KBBF, b) *C*2‐BBF, and c) Zn_2_BO_3_(OH) viewed along the *b*‐axis. d,e) Topologic graphs of [Be_2_BO_3_F_2_]_∞_ (left) and [Zn_2_BO_3_(OH)_2_]_∞_ (right) layers. f) The near parallel [BO_3_]^3−^ groups in the [Zn_2_BO_3_(OH)_2_]_∞_ layers.

The optical and thermal performances were characterized. As is shown in **Figure** **2**a, Zn_2_BO_3_(OH) exhibits a wide transparency window from UV to near infrared (NIR) region, while its UV cutoff edge reaches 204 nm with a reflectance rate of 9.8%, which satisfies the requirement of the UV optical applications. The transformed absorptance curve and the deduced bandgap of 5.33 eV were shown in the inset in Figure 2a. Thermogravimetric (TG) and differential scanning calorimetry (DSC) of Zn_2_BO_3_(OH) were presented in Figure 2b. There is no obvious weight loss or endothermic peak from room temperature to 440 °C, which indicates that Zn_2_BO_3_(OH) exhibits good thermal stability up to 440 °C. In the continuous heating procedure, an endothermic peak at 545 °C is observed in the region of 440–550 °C due to the release of hydroxyl in Zn_2_BO_3_(OH), which is in accordance with the theoretical weight loss of 6.3%. As shown in Figure 2c, Zn_2_BO_3_(OH) exhibits a SHG response of about 1.5 times that of KDP at the largest particle size range of 150–200 µm. And, the result also indicates that Zn_2_BO_3_(OH) is phase matchable at 1064 nm fundamental light based on the Kurtz–Perry rule.^16^

**Figure 2:**
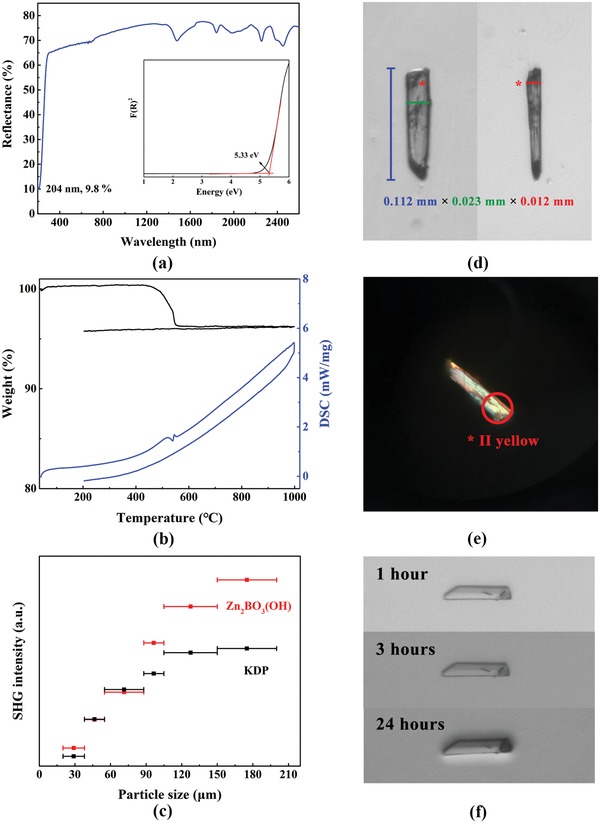
a) UV–vis–NIR and transformed absorptance curves. b) TG/DSC curves. c) Powder SHG measurements at 1064 nm. d,e) Crystals for the birefringence determination and its interference color observed in the cross‐polarized light. * is the same position of the crystal. f) The Zn_2_BO_3_(OH) crystal soaking in the water.

The birefringence and water‐resistant experiment of Zn_2_BO_3_(OH) were done based on two crystals. A wedge‐shaped crystal of Zn_2_BO_3_(OH) with the size of 0.112 mm × 0.023 mm × 0.012 mm (Figure 2d) was adopted for the birefringence measurement by using the cross‐polarizing microscope. The maximum interference color of second order yellow was observed in cross‐polarized light (Figure 2e) and matched with the Michal–Levy chart.^17^ The birefringence of Zn_2_BO_3_(OH) in the visible light region was calculated by the equation of

(1) R=Δn×d

where *R* is the retardation, Δ*n* is the birefringence, and *d* is the thickness. According to the results, the retardation is about 800 nm, which is corresponding to Δ*n* of about 0.067 in the visible region. This value is larger than those of commercial UV crystals like LBO, CBO, and CLBO. Since the crystal is too small to determine its orientation, the largest birefringence of Zn_2_BO_3_(OH) should be equal to or larger than 0.067, which also indicates that Zn_2_BO_3_(OH) is probably phase matchable for UV laser output. Another crystal was selected for the water‐resistant ability test. As shown in Figure 2f, the shape of this crystal was unaltered with water soaking for 1, 3, and 24 h, respectively, which suggests that Zn_2_BO_3_(OH) exhibits well water‐resistant ability and environmental stability.

The partial densities of states (PDOSs) of Zn_2_BO_3_(OH) are shown in **Figure** **3**a. The electronic states near the bandgap are mainly composed of 2*p* orbitals of the O atoms and 3d orbitals of the Zn atoms at the top of valence bands (VB), while 3*d* and 4*s* orbitals of the Zn atoms occupy the bottom of conduction bands (CB). Considering that the optical response of a crystal in the UV region originates mainly from the electronic transitions between the VB and CB states that are close to the bandgap, the [BO_3_]^3−^ and [ZnO_4_]^6−^ groups mainly determine the optical properties of Zn_2_BO_3_(OH). The calculated bandgap using generalized gradient approximation (GGA) functional is 3.10 eV for Zn_2_BO_3_(OH). Since GGA usually underestimates the bandgap,^18^ a scissor operator (2.23 eV) was adopted to shift the conduction bands to agree with the bandgap values of experiments. Further, the refractive indices of Zn_2_BO_3_(OH) were calculated and shown in Figure S3 (Supporting Information), which suggests that the birefringence of Zn_2_BO_3_(OH) is 0.0793@532 nm. The result is in accordance with the experimental one. Based on the calculated dispersion curve (Figure S4, Supporting Information), the shortest phase‐matching wavelength of 248 nm was estimated, which indicates that Zn_2_BO_3_(OH) is phase matchable for the 266 nm laser output.

**Figure 3:**
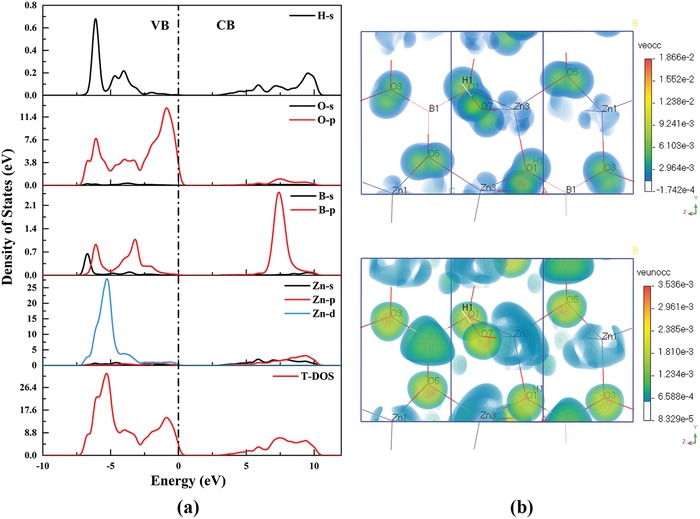
a) Partial and total DOS for Zn_2_BO_3_(OH). b) Occupied and unoccupied states of the VE process for Zn_2_BO_3_(OH).

The NLO properties were calculated based on the so‐called length‐gauge formalism derived by Aversa and Sipe, and lately developed by our group.^19^ According to the Kleinman approximation of point group 2, there are solely four nonzero NLO coefficients, *d*_14_ = *d*_25_ = *d*_36_ = 0.37 pm V^−1^, *d*_16_ = *d*_21_ = 0.60 pm V^−1^, *d*_23_ = *d*_34_ = 0.41 pm V^−1^, and *d*_22_ = −0.95 pm V^−1^. The calculated values are slightly larger than the experimental result measured by Kurtz–Perry method (1.5 × KDP, *d*_36_ = 0.39 for KDP). In order to analyze the contribution of NLO‐active electron states and units, the SHG‐weighted electron density analysis was adopted. The largest tensor *d*_22_ in the effective NLO coefficient *d*_eff_ is analyzed. The SHG process contains two virtual transition processes, namely, virtual electron (VE) and virtual hole processes. Based on the calculation results, VE domains the SHG process with a ratio of 89.3%. Therefore, the SHG process of VE with occupied and unoccupied states was examined (Figure 3b). It can be seen that the contributions of the SHG responses to *d*_22_ are mainly derived from the [BO_3_]^3−^ groups in the occupied and unoccupied states, while the Zn atoms also contribute to the SHG responses due to the distorted [ZnO_4_]^6−^ tetrahedra. It indicates that the [BO_3_]^3−^ and [ZnO_4_]^6−^ groups synergistically determine the SHG response of Zn_2_BO_3_(OH).

Owing to the conflicts between the beneficial paralleled [BO_3_]^3−^ units and layer tendency in borate system, the growth habits of UV and DUV NLO materials attracted a lot of attention.[qv: 8,13b] The previous views deem that the layered growth habit is caused by weak interlayer forces and large interlayer distances.^9^, ^20^ Inspired by the research on the anisotropy growth rate,^21^ we notice that the layer tendency for KBBF‐type structures is a result of the overlarge growth‐rate differences between inter‐ and intralayers. The interactions of the intralayers also need to be considered while assessing the growth habits. In order to evaluate the growth habits of the layered‐structural NLO crystals, a method from the perspective of released energy and growth rate was proposed. The released energy of the single layer was evaluated by the cohesive energy (CE), which is defined as the energy from the ground‐state free atoms to solid state matters. And, the binding energy (BE) was calculated for the released heat of interlayered combinations during the crystallization. And, the ratio of CE and BE was calculated to synergistically describe the anisotropy of crystal growth. For assessing the ratio in the same scale, the quantities of atom involved were also considered. In this method, the average CE/BE ratio is expected to near 1 for nonlayered growth habit. **Table** **1** lists the calculated values for KBBF, *C*2‐BBF, and Zn_2_BO_3_(OH), respectively. The details of the calculation method were presented in the Supporting Information. These three compounds give the CE values of −58.310, −56.250, and −92.573 eV and the BE values of −1.699, −5.305, and −4.009 eV, respectively. As for the values of CE/BE per atom, KBBF gives the largest value of 4.290, while *C*2‐BBF and Zn_2_BO_3_(OH) give values of 1.515 and 1.443, respectively, which suggests that KBBF has a largest anisotropic growth rate between the intra‐ and interlayers, and Zn_2_BO_3_(OH) and *C*2‐BBF show more balanced growth rates from the thermodynamic point of view.

**Table 1:** Thermodynamics anisotropy of crystal growth for KBBF, *C*2‐BBF, and Zn_2_BO_3_(OH)

Crystal	CE	BE	Quantities of atom	CE/BE (per atom)
KBBF	−58.310	−1.699	8	4.290
*C*2‐BBF	−56.250	−5.305	7	1.515
Zn_2_BO_3_(OH)	−92.573	−4.009	16	1.443

In conclusion, a beryllium‐free KBBF member, Zn_2_BO_3_(OH) was successfully synthesized. It features the same topological layer with KBBF by replacing [BeO_3_F]^5−^ to nontoxic [ZnO_3_(OH)]^5−^. Zn_2_BO_3_(OH) exhibits a SHG response of about 1.5 × KDP@1064 nm with the UV cutoff edge of 204 nm. The birefringence of Zn_2_BO_3_(OH) in the visible region was determined with the value of 0.067 which is larger than those of commercial UV crystals LBO, CBO, and CLBO. Additionally, Zn_2_BO_3_(OH) has excellent thermal and water‐resistant stabilities. Owing to the removal of interlayer cations, Zn_2_BO_3_(OH) shows better growth habits than that of KBBF. From the perspective of thermodynamics, the anisotropy of crystal growth for Zn_2_BO_3_(OH), i.e., the ratio of CE and BE was estimated, which is evidently smaller than that for KBBF, indicating an improved growth habit. We believe that the method can be a reliable approach for assessing the growth habit of layered‐structural crystal. Further experiments and the crystal growth are in progress.

## Experimental Section

*Synthesis*: ZnO (Tianjin Hengxing Chemical Reagent Co., Ltd., 99.5%), H_3_BO_3_ (Tianjing Baishi Chemical Reagent Co., Ltd., 99.5%), and KF·2H_2_O (Tianjing Hengxing Chemical Reagent Co., Ltd., 99.5%) were used as received. Crystals of Zn_2_BO_3_(OH) were synthesized from a hydrothermal reaction of 0.0814 g (0.001 mol) of ZnO, 0.3710 g (0.006 mol) of H_3_BO_3_, and 0.3765 g (0.004 mol) of KF·2H_2_O in a heated–sealed fluorinated ethylene propylene Teflon pouch.^22^ Four pouches and 30 mL distilled water were added in a Teflon‐lined autoclave together and then heated at 210 °C for 96 h and cooled to the room temperature with the rate of 2 °C h^−1^. The samples obtained from the pouches were washed by distilled water for removing the most of impurities. Colorless and block‐like crystals of Zn_2_BO_3_(OH) were further selected from the samples for separating out of the residual minor phase of ZnO. Finally, the pure sample of Zn_2_BO_3_(OH) was prepared with a yield of about 80% based on Zn.

*Single Crystal and Powder X‐Ray Diffraction*: Single crystal X‐ray diffraction data were collected from a crystal of Zn_2_BO_3_(OH) with dimensions of 0.174 mm × 0.137 mm × 0.128 mm. The structural data were collected by a Bruker SMART APEX II charge coupled device single‐crystal diffractometer equipped monochromatic Mo Kα radiation (λ = 0.71073 Å) at 293 K, and obtained data were integrated with a SAINT program.^23^ Programs from the SHELXTL crystallographic software package were used for calculations.^24^ All the nonhydrogen atoms were solved by direct methods and refined by full‐matrix least‐square techniques with anisotropic thermal parameters. Hydrogen atoms were added by the geometrical method. Final least‐square refinement was on *F*_o_^2^ with data having *F*_o_^2^ ≥ 2σ (*F*_o_^2^). The missing symmetry elements were checked with PLATON.^25^ Crystallographic data and structural refinement information for Zn_2_BO_3_(OH) were listed in Table S1 (Supporting Information). The refined atomic positions and equivalent isotropic displacement parameters were listed in Table S2 (Supporting Information). Selected bond lengths and angles, and anisotropic displacement parameters of Zn_2_BO_3_(OH) were listed in Tables S3 and S4 (Supporting Information), respectively.

Powder X‐ray diffraction (PXRD) data were collected on a Bruker D2 ADVANCE X‐ray diffractometer with monochromatic Cu Kα radiation (λ = 1.5418 Å). The PXRD pattern was recorded from 10° to 70° (2θ) with the scan step width of 0.02° and the rate of 1 s per step. Theoretical simulation pattern of Zn_2_BO_3_(OH) was conducted from the single crystal crystallographic data. The PXRD pattern matched well with the theoretical simulation one, as shown in Figure S1 (Supporting Information), which confirmed the sample purity of Zn_2_BO_3_(OH).

*Spectroscopy*: UV–vis–NIR diffuse reflectance spectrum of Zn_2_BO_3_(OH) was measured on a Shimadzu SolidSpec‐3700 DUV spectrophotometer at room temperature. The data were collected in the wavelength range of 200–2600 nm and converted to absorptance with the Kubelka–Munk equation.^26^ Infrared (IR) spectrum data were collected from a Shimadzu IR Affinity‐1 Fourier transform infrared spectrometer in the range of 400–4000 cm^−1^ using KBr pellets (Figure S2, Supporting Information).

*Thermal Analysis*: TG and DSC of Zn_2_BO_3_(OH) were performed on a simultaneous NETZSCH STA 449 F3 thermal analyzer instrument with flowing N_2_ atmosphere. The sample was enclosed in a Pt crucible and heated from 25 to 1000 °C with a rate of 10 °C min^−1^.

*SHG Measurements*: Powder SHG measurement at 1064 nm was carried out by using Kurtz–Perry method^16^ with a Q‐switched Nd:YVO_4_ solid‐state laser. Polycrystalline Zn_2_BO_3_(OH) samples were ground and sieved into the following particle size ranges: 20–38, 38–55, 55–88, 88–105, 105–150, and 150–200 µm. The sieved KDP samples were used as the reference for SHG measurements.

*Microscopy*: The birefringence and water‐resistant ability of Zn_2_BO_3_(OH) were observed on a SHANGHAI YIYUAN polarizing microscope YYP‐600E with visible light filter.

*Computation Methods*: To further investigate the relationships between the optical performances and crystal structure of Zn_2_BO_3_(OH), the electronic structure calculations based on density functional theory (DFT) were adopted.^27^ The first principle calculations were performed by the plane‐wave pseudopotential method implemented in the CASTEP, package based on DFT with the norm‐conserving pseudopotential.^28^ The exchange–correlation functional was Perdew–Burke–Ernzerhof^29^ functional within the GGA. The plane‐wave energy cutoff was set at 830.0 eV. The valence electrons adopted for calculations were H‐1*s* ^1^, B‐2*s* ^2^ 2*p* ^1^, O‐2*s* ^2^ 2*p* ^4^ and Zn‐3*d* ^10^ 4*s* ^2^, respectively. The *k*‐point separation was set as 0.05 Å^−1^ in the Brillouin zone, resulting in the corresponding Monkhorst–Pack *k*‐point meshes of 3 × 3 × 2.^30^ The empty bands were set as 3 times of valence bands in the calculation to ensure the convergence of optical properties. The scissor operator was adopted to shift the conduction bands to agree with the bandgap values of the experimental result. The NLO properties were calculated based on the so‐called length‐gauge formalism derived by Aversa and Sipe,^19^ which was successfully applied in the calculations of many NLO crystals such as KBBF, LBO, and β‐BBO. The SHG‐weighted electron density analysis was adopted to explore the origin of the NLO properties of Zn_2_BO_3_(OH).

*Thermodynamic Anisotropy of Crystal Growth*: The morphology of a crystal is the synergistical results of thermodynamic and kinetic factors. A lot of parameters such as the objective structures, temperature, pressure, and ion transportation play important roles during the crystallization procedure. First and foremost, the crystal structure could be regarded as the intrinsic character that influenced the growth habit. In the past years, several assessment methods for evaluating the interlayer forces based on the interlayer distances or Coulomb's law were designed and carried out. The former method evaluated interlayer forces by the distances of the adjacent layers. The latter method calculated the interlayered forces via the interactions of adjacent atoms. In the previous work, a method for evaluating the interaction of adjacent layers by binding energies based on DFT was proposed. According to the previous research provided by Sun and co‐workers,^21^ crystallization induced the decrease of Gibbs free energy and chemical potential. And, the released bonding energy was proportional to the growth rate. Accordingly, the anisotropic growth habit was presented via the energetic differences of the intra‐ and interlayers.

Thermodynamic crystal growth anisotropy was calculated on KBBF, *C*2‐BBF, and Zn_2_BO_3_(OH) due to their similar topological layers. The modeling and calculations were carried out by the following steps – 1) the energy of ground‐state atoms including K, Zn, Be, B, O, F, and H in a 15 Å × 15 Å × 15 Å unit cell was calculated, respectively; 2) the energies of the single layer and crystal for each compound were calculated; 3) BE was calculated according to the differences between the energies of the single layer and crystals; 4) CE was calculated according to the differences between the energies of the single layer and ground‐state atoms; 5) the ratio of CE and BE per atom was calculated for assessing the thermodynamic crystal growth anisotropy. All the single layer and double layers were placed in a vacuumed layer with the thickness of 15 Å for avoiding the influences between each other.

## Conflict of Interest

The authors declare no conflict of interest.

## Supporting information

Supplementary
