# Supplementary material for: Nontoxic KBBF Family Member Zn2BO3(OH): Balance between Beneficial Layered Structure and Layer Tendency
Source: Adv Sci (Weinh). 2019 Sep 16;6(22):1901679. doi: 10.1002/advs.201901679 (PMC6864501; doi:10.1002/advs.201901679)

Copyright WILEY-VCH Verlag GmbH & Co. KGaA, 69469 Weinheim, Germany, 2019.

Supporting Information

for *Adv. Sci.,* DOI: 10.1002/advs.201901679

Nontoxic KBBF Family Member $Zn_2BO_3(OH)$: Balance between Beneficial Layered Structure and Layer Tendency

*Xuefei Wang, Fangfang Zhang, Le Gao, Zhihua Yang, and Shilie Pan**

Copyright WILEY-VCH Verlag GmbH & Co. KGaA, 69469 Weinheim, Germany, 2019.

Supporting Information

Nontoxic KBBF Family Member $Zn_2BO_3(OH)$: Balance between Beneficial Layered Structure and Layer Tendency

*Xuefei Wang, Fangfang Zhang, Le Gao, Zhihua Yang and Shilie Pan**

Table S1. Crystal data and structure refinement of $Zn_2BO_3(OH)$.

Empirical formula	$Zn_2BO_3(OH)$
Formula weight	206.56
Wavelength (Å)	0.71073
Temperature (K)	296.15
Crystal system	Monoclinic
Space group	$P2_1$ (No. 4)
a / Å	5.730(7)
b / Å	4.952(4)
c / Å	6.881(6)
β / °	99.09(5)
Volume /Å^3	192.8(3)
Z, ρ_{calcd} / g·cm^{-3}	2, 3.559
μ / mm^{-1}	12.302
F(000)	196
Crystal size / mm^3	0.174 × 0.137 × 0.128
Theta range for data collection	2.998 to 24.997
Limiting indices	$-5 \le h \le 6$, $-5 \le k \le 5$,

	$-7 \le l \le 8$
Reflections collected / unique	980 / 647 [R_{int} = 0.0250]
Completeness / %	97
Data / restraints / parameters	647 / 8 / 68
Goodness-of-fit on F^2	1.057
Final *R* indices [I>2sigma(I)] [a]	$R_1 = 0.0347$, $wR_2 = 0.0599$
R indices (all data) [a]	$R_1 = 0.0470$, $wR_2 = 0.0667$
Absolute structure parameter	0.08(9)
Largest diff. peak and hole/ e·Å^{-3}	0.69 and –0.90

[a] $R_1 = \Sigma||F_o| - |F_c||/\Sigma|F_o|$ and $wR_2 = [\Sigma w\text{-}(F_o^2 - F_c^2)^2/ \Sigma wF_o^4]^{1/2}$ for $F_o^2 > 2\sigma(F_o^2)$.

Table S2. Selected bond distances (Å) and angles (º).

Zn1 - O2$^{\#2}$	1.929(10)	O2$^{\#2}$ - Zn1 - O2	107.7(3)
Zn1 - O2	1.952(9)	O2$^{\#2}$ - Zn1 - O3$^{\#3}$	109.7(4)
Zn1 – O3$^{\#3}$	1.981(10)	O2 - Zn1 - O3$^{\#3}$	108.0(4)
Zn1 - O4	1.948(9)	O2$^{\#2}$ - Zn1 - O4	112.0(4)
Zn2 - O1	1.973(9)	O4 - Zn1 - O2	114.0(6)
Zn2 - O1$^{\#4}$	1.989(10)	O4 - Zn1 - O3$^{\#3}$	105.2(5)
Zn2 - O3$^{\#5}$	1.939(10)	O1 - Zn2 - O1$^{\#5}$	104.8(3)
Zn2 - O4	1.939(9)	O3$^{\#6}$ - Zn2 - O1$^{\#5}$	104.8(4)
B1- O1	1.360(19)	O3$^{\#6}$ - Zn2 - O1	107.8(4)
B1 - O2$^{\#6}$	1.348(18)	O3$^{\#6}$ - Zn2 - O4	126.4(5)
B1 - O3	1.420(3)	O4 - Zn2 - O1	108.3(6)
		O4 - Zn2 - O1$^{\#5}$	102.6(4)
		O1 - B1 - O3	117.0(14)
		O2$^{\#4}$ - B1 - O1	125.0(2)
		O2$^{\#4}$ - B1 - O3	118.4(14)

Symmetry transformations used to generate equivalent atoms:

#1) 1+x, y, z; #2) 2-x,1/2+y,1-z; #3) 1+x,1+y, z; #4) -1+x, y, z; #5) 1-x, 1/2+y, 2-z; #6) x, 1+y, z

Table S3. Atomic coordinates, equivalent isotropic displacement parameters (Å) and bond valence sums (BVS).

Atom	x	y	z	U_{eq}[a]	BVS[b]
Zn1	10553(2)	4121(3)	6446(2)	13.5(4)	2.05
Zn2	5718(2)	4295(4)	8727(2)	12.5(4)	2.01
B1	3210(20)	-760(60)	7102(18)	9(3)	2.97
O1	4653(16)	502(17)	8581(14)	19(2)	1.98
O2	11518(16)	481(17)	5827(14)	19(2)	2.12
O3	3427(17)	-3604(19)	6975(16)	20(3)	1.88
O4	9119(14)	4340(30)	8827(12)	19(2)	1.05

[a]U_{eq} is defined as one-third of the trace of the orthogonalized U_{ij} tensor.

[b]Bond valence sums are calculated by using the bond-valence model ($S_i = \exp[(R_o - R_i)/B]$, where R_o and B are bond valence parameters, and R_i is the length of bond.

Figure S1. PXRD patterns of $Zn_2BO_3(OH)$.

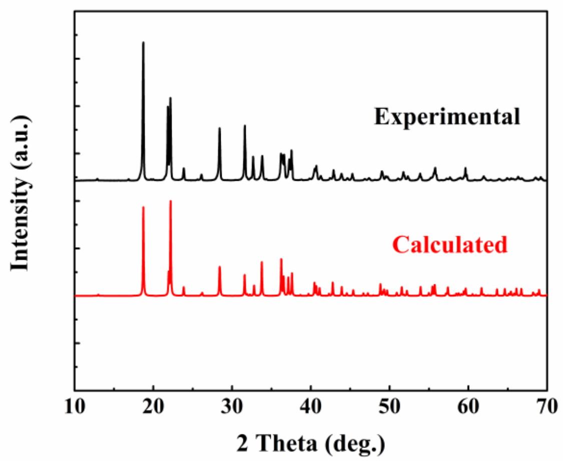

Figure S2. IR spectrum. The presence of $[OH]^-$ was confirmed by the board peaks at 3366 and 3407 cm^{-1}. The absorption peaks from 1500 - 800 cm^{-1} can be assigned as the asymmetric stretching vibrations and symmetric stretching vibrations of $[BO_3]^{3-}$ while the peaks in the range of 400-800 cm^{-1} also confirmed the bending vibrations of $[BO_3]^{3-}$. These results are in accordance with previous report of other borate.

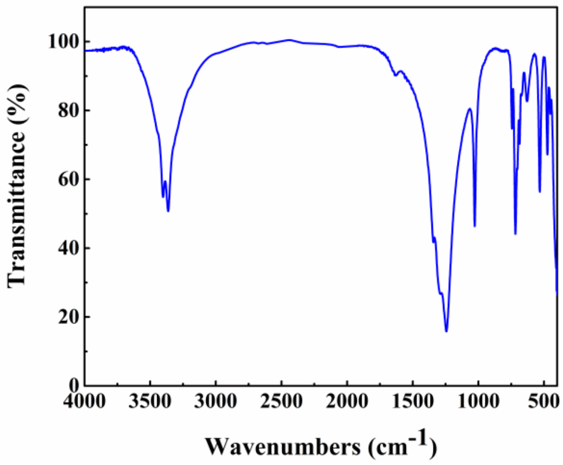

Figure S3. Elemental analysis. Energy dispersive X-ray spectroscopy (EDX) was performed to confirm the elements in the solved structure. The EDX analysis confirms the existence of Zn, B and O and excludes other elements.

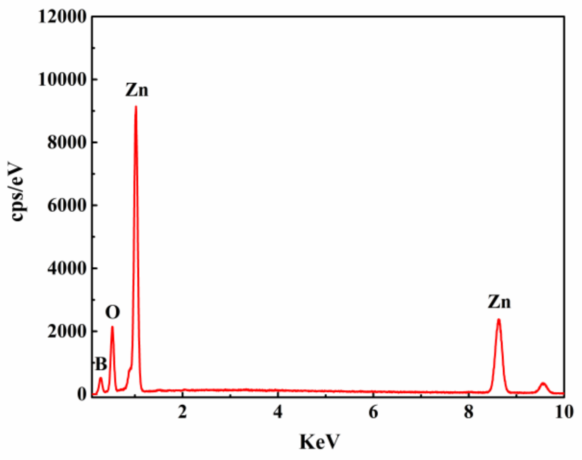

Figure S4. Calculated refractive indices. The axis system in which the refractive indices follow the convention: $n_z > n_y > n_x$ was used. The calculated refractive indices display the chromatic dispersion curves. As $n_z - n_y < n_y - n_x$, it depicts that $Zn_2BO_3(OH)$ is a negative biaxial crystal. And the calculated birefringence is 0.0793 @ 532 nm. According to the curves, the shortest phase-matching second-harmonic wavelength of 248 nm was estimated.

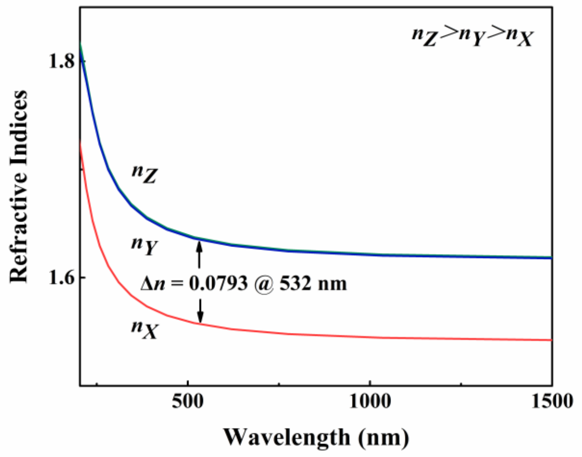

Supplement: Supplementary file 1 — Supplementary [file ADVS-6-1901679-s001.pdf]
